# Pneumomédiastin compliquant une crise d’éclampsie: à propos d'un cas

**DOI:** 10.11604/pamj.2014.19.236.5250

**Published:** 2014-10-31

**Authors:** Mouhssine Doumiri, Youssef Motiaa, Nezha Oudghiri, Anas Tazi Saoud

**Affiliations:** 1 Service d'Anesthésie-Réanimation, Hôpital Maternité Souissi, Centre Hospitalier Universitaire, Rabat, Maroc

**Keywords:** Pneumomediastin, éclampsie, état de mal convulsif, grossesse, Pneumomediastinum, eclampsia, status epilepticus, pregnancy

## Abstract

Le pneumomédiastin associé à l'emphysème sous cutané et le pneumothorax, sont des complications rares de la grossesse et surviennent au cours du travail obstétrical. Nous rapportons l'observation d'une parturiente de 25ans, sans antécédent pathologique particulier, admise pour une crise d’éclampsie à 36 semaines d'aménorrhées avec une mort fœtale et trouble de la conscience. L'examen clinique a montré un emphysème sous cutané étendu du visage jusqu’à l'abdomen sans notion de traumatisme et un score de Glasgow à 10. Après mise en condition, traitement de la crise d’éclampsie, stabilisation de la tension artérielle et retour à l’état de conscience, une TDM cervico-thoraco-abdominale a été demandée et a révélé la présence d'un pneumomédiastin important avec un discret pneumothorax droit postérieur et un pneumopéritoine important qui n'ont pas nécessité de drainage pleural. Deux jours après son admission, la patiente a expulsé un mort-né d'un poids de 1800 grammes avec forceps et sans efforts d'expulsions sous analgésie péridurale. Le contrôle radiologique à une semaine a noté une nette diminution de l'emphysème sous cutané et du pneumomédiastin. La patiente a quitté l'hôpital après dix jours.

## Introduction

Le pneumomédiastin associé à l'emphysème sous cutané et le pneumothorax, sont des complications rares de la grossesse, survenant surtout au cours du travail obstétrical décrivant le Hamman's Syndrome [1]. Les complications respiratoires liées aux crises d’éclampsie sont surtout dues à l'inhalation du contenu gastrique et l’œdème pulmonaire neurogénique [2]. Nous rapportons l'observation d'une parturiente admise pour une crise d’éclampsie à 36 semaines d'aménorrhées compliquée d'un pneumomédiastin associé à un emphysème sous cutané.

## Patient et observation

**Figure 1:**
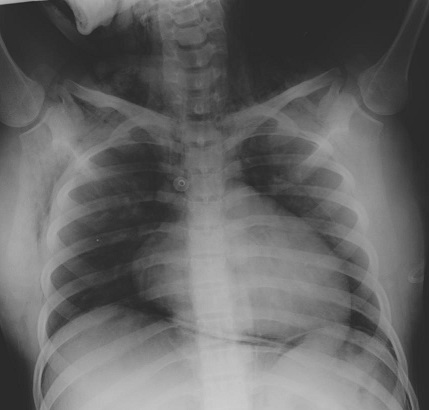
Radio pulmonaire de face montrant un pneumomédiastin et un emphysème sous cutané

**Figure 2:**
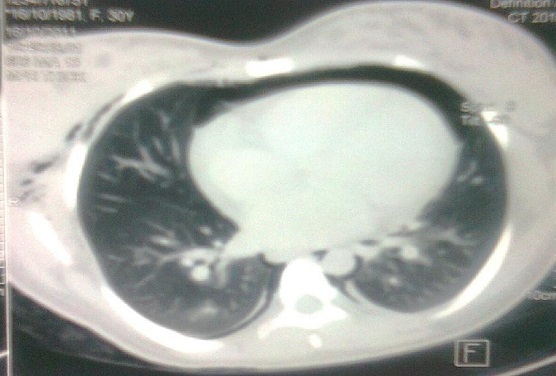
Scanner thoracique montrant un pneumomédiastin associé à un discret pneumothorax droit et un emphysème sous cutané

**Figure 3:**
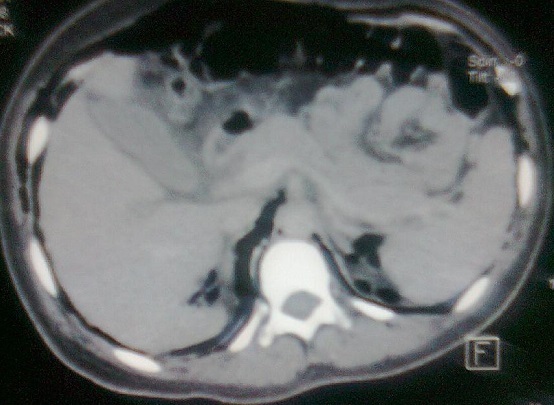
Scanner abdominale montrant un pneumopéritoine et un emphysème sous cutané

Madame K, âgée de 25 ans, primipare, sans antécédents particuliers, admise aux urgences pour crise d’éclampsie sur une grossesse de 36 semaines d'aménorrhées. L'examen à l'admission a trouvé un score de Glasgow à 10, sans déficit neurologique sensitivomoteur, une pression artérielle à 185 / 120 mmHg, une fréquence cardiaque à 120 battements par minutes, une fréquence respiratoire à 20 cycles /min et une SpO2 à 95% à l'air ambiant, un emphysème sous cutané étendu du visage jusqu′à l′abdomen avec une diminution des murmures vésiculaires au champ pulmonaire droit et sans signe de traumatisme. L'examen obstétrical a montré des bruits cardiaque fœtaux négatifs et un col long fermé et postérieur sans saignement ni contracture utérine. La confirmation d'une grossesse non évolutive de 36 semaines d'aménorrhées a été faite par une échographie obstétricale. Après mise en condition: oxygénothérapie, décubitus latérale gauche, un traitement de la crise d’éclampsie par le sulfate de magnésium (4g bolus en 20min puis relais par perfusion continue d’ 1g par heure) et un traitement de l'hypertension artérielle par la nicardipine( 0,5mg/ heure en perfusion continue). Après une heure, la patiente s'est réveillée et devenue consciente avec un GCS à 15. Une radio pulmonaire de face a montré un pneumomédiastin et un emphysème sous cutané (Figure 1). Une TDM cervico-thoraco-abdominale a révélé la présence d'un pneumomédiastin important, un discret pneumothorax postérieur droit et un important pneumopéritoine (Figure 2, Figure 3). Le bilan biologique (NFS, ionogramme sanguin, bilan hépatique, TP et TCA) est revenu normal. Deux jours après son admission, la patiente a expulsé par voie vaginale un mort-né d'un poids de 1800 grammes avec forceps et sans efforts d'expulsions sous analgésie péridurale. Le contrôle radiologique à une semaine a noté une nette diminution de l'emphysème sous cutané et du pneumomédiastin. La patiente a quitté l'hôpital au dixième jour.

## Discussion

Le pneumomédiastin spontané se voit rarement dans la pratique obstétricale. Seuls 200 cas approximativement ont été décrits dans le monde [3]. Il se constitue le plus souvent durant la seconde phase de travail décrivant le Hamman's Syndrome, responsable des douleurs thoraciques, de dyspnée, de cyanose et d'hypotension. Dans notre cas, la symptomatologie décrite était en dehors du travail et consécutive à la crise convulsive tonico-clonique de l’éclampsie, Le pneumomédiastin avec pneumothorax compliquant une crise convulsives tonico-cloniques et extrêmement rare, seul deux cas sont décrits dans la littérature [4, 5]. Le mécanisme serait la conséquence d'un gradient de pression entre les alvéoles et l'interstitium pulmonaire suite à une manœuvre de Valsalva contre une glotte fermée, il en résulte une augmentation de la pression intra-alvéolaire, puis une rupture des alvéoles avec libération d'air à travers les tissus péribronchiques dans le médiastin, le hile et les tissus sous-cutanés. En cas de rupture alvéolaire directement dans la cavité pleurale, il en résulte un pneumothorax [4, 5]. La survenue d'un emphysème sous cutané chez la parturiente au cours du travail ou après une crise convulsive impose un scanner cervico-thoraco-abdominal. Il permet de confirmer le diagnostic et de chercher une cause sous jacente (bulle d'emphysème, kyste aérique pulmonaire). L’évolution se fait vers une rémission complète du pneumomédiastin et la régression de l'emphysème sous-cutané en quelques jours. La gestion du pneumomédiastin au cours du travail repose sur l'oxygénothérapie, l'analgésie péridurale et rarement le drainage pleural est indiqué. L'utilisation d'un forceps devrait diminuer les efforts expulsifs de la femme durant la seconde phase du travail. Dans le cas où une césarienne est indiquée chez une patiente ayant présenté ce syndrome, l'anesthésie locorégionale sera certainement préférable, afin d’éviter la ventilation en pression positive qui risque d'aggraver un éventuel pneumothorax [6].

## Conclusion

L'aggravation du pneumomédiastin associé à un pneumothorax est surtout en péripartum. Elle est liée aux efforts expulsifs du travail avec fermeture de la glotte pour pousser et à l'anesthésie générale pour césarienne. Dans ce cas un drainage pleural est systématique avant l'induction. En générale, Le pronostic de ces complications reste bon.
